# Health Disparities in Central Line-Associated Bloodstream Infections: Analysis of the U.S. National Inpatient Sample Database (2016–2022)

**DOI:** 10.3390/idr17050105

**Published:** 2025-08-28

**Authors:** Nicholas Mielke, Ryan W. Walters, Faran Ahmad

**Affiliations:** 1 Department of Medicine, Creighton University School of Medicine, 7710 Mercy Road, Suite 202, Omaha, NE 68124, USA; 2 Department of Clinical Research and Public Health, Creighton University School of Medicine, Omaha, NE 68124, USA; 3 Division of Infectious Diseases, Creighton University School of Medicine, Omaha, NE 68124, USA

**Keywords:** central line-associated bloodstream infections, health disparities, National Inpatient Sample, hospital cost, in-hospital mortality

## Abstract

Introduction: Central line-associated bloodstream infections (CLABSIs) are a major cause of morbidity and mortality, yet health disparities in CLABSI incidence and outcomes remain understudied. This study evaluates these disparities and their impact on CLABSI rates, in-hospital mortality, hospital length of stay (LOS), and costs using the National Inpatient Sample (NIS) from 2016 to 2022. Methods: We conducted a retrospective analysis of adult hospitalizations using the NIS database that included central venous catheter placement and identified CLABSI using AHRQ’s Patient Safety Indicator 07. Primary outcomes included CLABSI incidence and in-hospital mortality; secondary outcomes were LOS and inflation-adjusted hospital costs. Outcomes were analyzed using logistic and lognormal regression models, focusing on demographic and clinical variables that included sex, race, socioeconomic status, and insurance type. Results: Among 11.5 million CVCs placed between 2016 and 2022, 6.56 million met CLABSI eligibility criteria, with 1 in 400 (0.25%) complicated by CLABSI. Blacks had 29.8% higher adjusted odds of CLABSI than Whites (*p* < 0.001), whereas Medicaid beneficiaries had 18.4% higher odds compared to those privately insured (*p* = 0.002). CLABSI was associated with a 97% increase in LOS and an 82% increase in hospital costs (both *p* < 0.001). In-hospital mortality was 13.3% and did not differ significantly by CLABSI status after adjustment. Discussion: Racial and socioeconomic disparities persist in CLABSI incidence and healthcare resource utilization, with Blacks and Medicaid beneficiaries at the highest risk. Although CLABSI rates returned to pre-pandemic levels in 2022, associated costs and LOS remained elevated. Further research and targeted prevention strategies are needed to reduce health disparities and improve patient outcomes.

## 1. Introduction

Central venous catheters (CVCs) are large catheters commonly placed in critical care settings to administer life-saving fluids, medications, and blood products. Past studies have suggested millions of CVCs are placed annually in the U.S. [1]. Complications can include infection, bleeding, cardiac arrhythmia, and thrombosis. Infections can remain localized around the catheter site or spread to the bloodstream, leading to central line-associated bloodstream infections (CLABSI) [1,2]. The National Healthcare Safety Network, which is part of the U.S. Centers for Disease Control and Prevention (CDC), defines CLABSI as “A laboratory confirmed bloodstream infection (BSI)” where an eligible BSI organism is identified, and an eligible central line is present on the laboratory confirmed bloodstream infection date of event or the day before [3]. CLABSIs affect more than 30,000 patients per year in the U.S. and carry a significantly increased risk for mortality [1,4,5]. In fact, a recent high-quality systematic review and meta-analysis estimate an overall CLABSI rate of 4.8 per 1000 catheter-days [1]. In response, numerous hospital-led and national initiatives for reducing CLABSI have been launched in recent years and continue to be implemented today because of their impact on other hospitalization-related outcomes as well, including hospital length of stay (LOS) and cost [6]. Healthcare-associated infections, particularly those associated with CVC use, are recognized as critical quality indicators and represent an international patient safety goal set forth by the Joint Commission International [7]. Additionally, CVC-related infection rates serve as important measures for healthcare quality assessment, influencing institutional practices and policies aimed at patient safety improvement [8].

According to the Joint Commission’s CLABSI toolkit published in 2013, factors such as age, sex, and underlying conditions increase the risk of CLABSI [9]. However, a closer examination of the literature reveals that some of the cited studies are limited. For instance, one study [10] was a small cohort, and another [11] focused solely on pediatric patients with peripherally inserted central catheters. More recent studies identify risk factors such as immunosuppression, total parenteral nutrition, COVID-19 infection, and the number of catheter days [12,13]. Despite these findings, no recent large-scale studies have thoroughly assessed the role of key risk factors for CLABSI in adults.

One important determinant that has been largely overlooked in the literature is healthcare inequities, which are defined as largely preventable differences in health outcomes adversely affecting populations facing greater barriers to optimal health. These inequities are closely linked to intergenerational social, economic, and environmental disadvantages—primarily affecting racial and ethnic minority groups and individuals with low socioeconomic status. Recent pediatric studies have demonstrated that significant racial and ethnic disparities exist in CLABSI rates, with Black and Hispanic children experiencing consistently higher infection rates than White children, and these disparities persist even after adjustment for known clinical risk factors, underscoring the role of systemic inequities in pediatric healthcare delivery [14,15,16]. These disparities—based on race, ethnicity, sex, and socioeconomic status—persist across the U.S. healthcare system and negatively affect outcomes in various conditions and interventions.

The primary objective of this study is to evaluate the impact of health disparities on the development of CLABSI in patients with CVCs using the National Inpatient Sample (NIS) database. Secondary objectives include examining the role of leading health indicators and further investigating how specific health disparities influence hospital-related outcomes among patients with CLABSI.

## 2. Methods

### 2.1. Ethics Statement

This study was acknowledged as not human subjects research by the Institutional Review Board at Creighton University (InfoEd record number: 2005058).

### 2.2. Data Source

This study used the 2016 to 2022 NIS, which is a 20% stratified sample of all discharges from community hospitals in the U.S. and is part of a family of databases within the Healthcare Cost and Utilization Project (HCUP) developed in a partnership with the Agency for Healthcare Research and Quality (AHRQ). When weighted, the NIS contains more than 35 million annual discharges and affords national-level estimates [17].

### 2.3. Study Cohort

The NIS does not provide a present-on-admission indicator for any diagnosis. As such, we identified hospitalizations eligible for hospital-acquired CLABSI using AHRQ’s patient safety indicator 07 [18,19]. Specifically, hospitalizations were included in this study if the patient (1) was 18 years of age or older, (2) had a CVC placed during the hospital stay, (3) had at least a two-day hospital LOS, (4) did not have CLABSI as the primary discharge diagnosis, (5) did not have a diagnosis of cancer or other immunosuppressed state, (6) did not have a major diagnostic category for newborn and other neonates (MDC 15), and (7) did not have an ungroupable Medicare-severity diagnosis-related group (MS-DRG 999). Patients included in this study had centrally inserted central venous catheters (CVCs); those with peripherally inserted central catheters (PICCs) or peripheral intravenous catheters were excluded. See Supplemental Table S1 for all ICD-10 diagnosis and procedure codes used in this study. From 2016 through 2022, there were an estimated 11,514,602 CVCs placed in U.S. community hospitals (unweighted: 2,302,921), of which an estimated 6,559,663 (unweighted: 1,311,933) were eligible to evaluate hospital-acquired CLABSI per AHRQ guidance (57.0%, 95% CI: 56.7% to 57.3%).

### 2.4. Outcomes

Primary outcomes included overall and year-over-year trends in CLABSI and all-cause in-hospital mortality. Secondary outcomes included LOS and hospital cost, that was inflation-adjusted to mid-year 2022 US dollars [20].

### 2.5. Health Disparities and Leading Health Indicators

Per the National Institute on Minority Health and Health Disparities (NIMHD), which defines populations that experience health disparities, we focused on stratifying patients by sex, race/ethnicity (White, Black, Hispanic, other), socioeconomic status via income quartile, and population size of home community [21]. Additionally, we investigated available data regarding the Leading Health Indicators, as per the U.S. Department of Health and Human Services Office of Disease Prevention and Health Promotion Healthy People 2030 initiative, which included primary payer (Medicare, Medicaid, private, other) and history of alcohol use disorder [22]. Medicare and Medicaid are government-sponsored health insurance programs in the United States; Medicare primarily covers individuals aged 65 and older and certain disabled populations, whereas Medicaid provides coverage to low-income individuals and families.

### 2.6. Covariates

For each hospitalization, we abstracted patient age, a count of the number of CVC’s placed during the hospital stay, whether the CVC was placed in the intensive care unit (ICU), need for invasive mechanical ventilation, vasopressor use, total parenteral nutrition, hemodialysis during the hospital stay, health-related social needs (employment, family, housing, psycho-social, socioeconomic), and comorbidity burden using the Charlson Comorbidity Index (CCI) [23]. Because the NIS does not include an indicator of ICU stay, we considered intubation and/or vasopressor use to indicate an ICU stay; a CVC was considered placed in the ICU if it occurred within two days of intubation or vasopressor use. We also abstracted treatment facility location/teaching status (rural, urban non-teaching, urban teaching).

### 2.7. Statistical Analysis

Continuous variables are presented as median, interquartile range, minimum, and maximum, whereas categorical variables are presented as percentages. Trend analyses were conducted using orthogonal polynomial contrasts in which we evaluated linear, quadratic, and cubic trends. Trends for in-hospital death were stratified by CLABSI status, with between-CLABSI status differences evaluated via year-by-CLABSI interaction effect. Demographic and clinical characteristics associated with CLABSI and in-hospital mortality were evaluated using logistic regression models, whereas LOS and inflation-adjusted hospital costs were evaluated using lognormal regression. For all outcomes, adjusted models included common health disparities (sex, race/ethnicity, income quartile, urban/rural status), leading health indicators (primary payer, alcohol abuse), and covariates that included patient age, income quartile, health-related social needs, CVC count, whether the CVC was placed in the ICU, CCI, facility location-teaching status, and year of hospitalization. The models for in-hospital mortality, LOS, and inflation-adjusted hospital cost included CLABSI status; we also added separate two-way interaction effects to the adjusted model to evaluate whether the effect of CLABSI differed across the common health disparities and leading health indicators. Although the purpose of our study was on effect sizes and signal, we report Harrell’s c-statistic for all adjusted logistic regression models. Further, because the NIS does not document care received or disposition for hospitalizations ending in transfer, we conducted a sensitivity analysis for in-hospital death that excluded hospitalizations in which the patient was transferred to another acute care facility to receive continued care. The functional forms of patient age and CCI were evaluated using restricted cubic splines with knot points at the 5th, 35th, 65th, and 95th percentiles; the decision to retain non-linear forms was informed by the likelihood ratio test [24]. An asterisk indicates that a result could not be presented per the NIS Data Use Agreement, as the unweighted hospitalization counts were 10 or fewer [25]. All analyses were conducted using SAS v. 9.4 (SAS Institute Inc., Cary, NC, USA), accounted for the NIS sampling design, and were weighted to provide national-level estimates. Two-tailed *p* < 0.05 was used to indicate statistical significance. Given that the purpose of our study was to quantify effect sizes for main effects and interaction effects, we decided *a priori* not to adjust for multiple comparisons.

## 3. Results

### 3.1. Baseline Demographic and Clinical Characteristics

Descriptive statistics for exclusions are provided in Supplemental Table S2. Demographic and clinical characteristics for included hospitalizations are provided in Table 1. Regarding demographics, the median patient age was 63, 48% were female, 69.3% were White, 32.2% were in the lowest income quartile, and 16.7% resided in a rural zip code. Clinically, 9.1% had multiple CVCs placed during the hospitalization, 31.2% had a suspected ICU stay, and 24.4% had their CVC placed in the ICU.

### 3.2. CLABSI Rate

**Figure 1:**
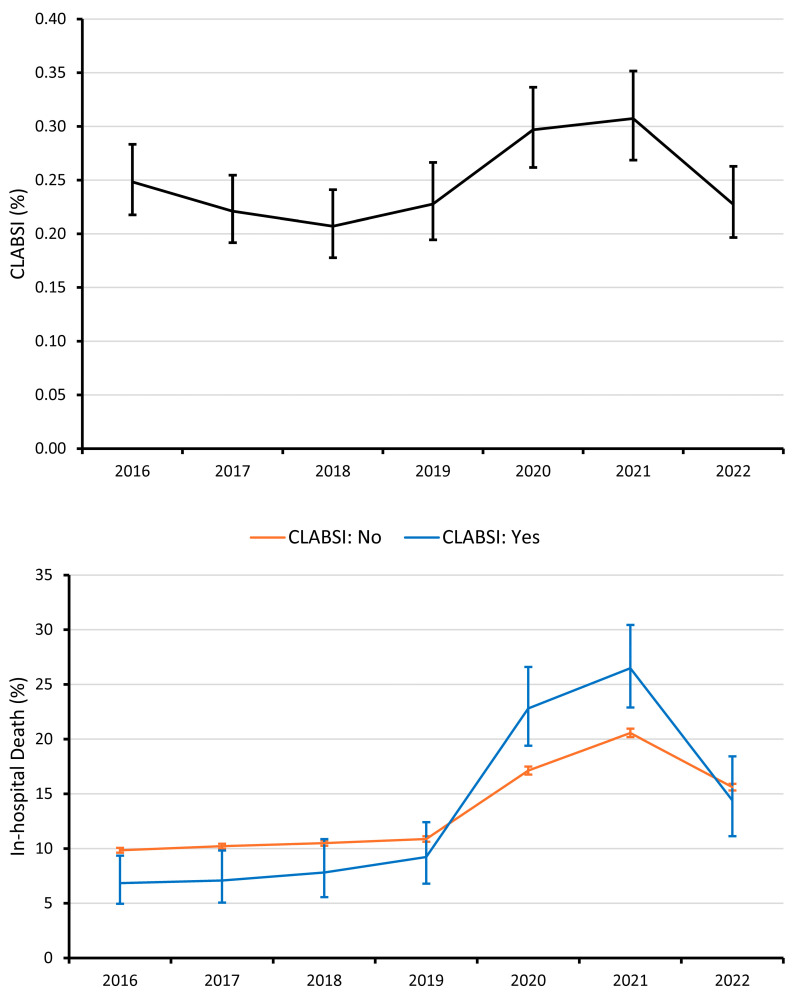
Year-over-year trend in CLABSI (**top**) and in-hospital death (**bottom**). Error bars represent 95% Agresti-Coull confidence intervals.

The overall rate of CLABSI during the study period was 0.25% (95% CI: 0.24% to 0.26%). As shown in Figure 1, the rate of CLABSI showed a cubic trend across the study period (cubic *p*_trend_ = 0.002) with CLABSI rates being statistically similar from 2016 through 2019 (all between-year differences *p* > 0.05), increasing during the COVID pandemic in 2020 and 2021 (*p* < 0.05 compared to all other years), before returning to a pre-COVID pandemic rate in 2022.

**Figure 2:**
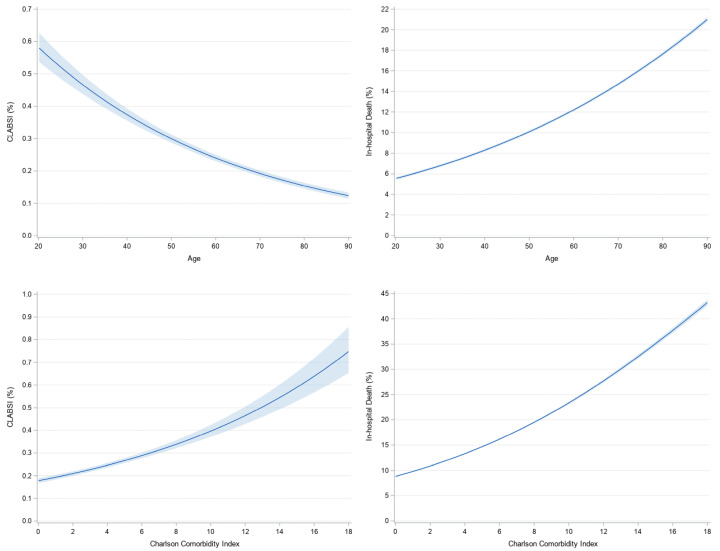
Unadjusted rate of CLABSI by age (**top**, **left**) and Charlson Comorbidity Index (**bottom**, **left**) as well as the unadjusted in-hospital death rate by age (**top**, **right**) and Charlson Comorbidity Index (**bottom**, **right**). The thick line presents the estimated CLABSI rate or in-hospital death rate for a specific age or comorbidity burden with shaded areas representing 95% confidence intervals.

Unadjusted and adjusted model results for CLABSI are presented in Table 2 and Figure 2. Overall, the adjusted model had good discriminant ability as Harrell’s c-statistic was 0.71. Specific to common health disparities and leading health indicators, Blacks averaged 29.8% greater adjusted odds of CLABSI compared to Whites (95% CI: 18.1% to 42.5%, *p* < 0.001). When compared to privately insured, Medicaid beneficiaries had 18.4% greater adjusted odds of CLABSI (95% CI: 6.3% to 31.9%, *p* = 0.002) and Medicare beneficiaries had 15.1% greater adjusted odds of CLABSI (95% CI: 3.1% to 28.5%, *p* = 0.023). Further, alcohol abuse was associated with 29.0% lower adjusted odds of CLABSI (95% CI: 17.9% to 38.5%, *p* < 0.001). For covariates, higher adjusted odds of CLABSI were associated with younger age, being treated at an urban teaching hospital, having additional CVCs placed during the hospital stay, having the CVC placed outside of the ICU, and having a greater comorbidity burden.

### 3.3. In-Hospital Mortality Rate

The overall in-hospital mortality rate during the study period was 13.3% (95% CI: 13.2% to 13.4%). As shown in Figure 1, the in-hospital mortality rate for both hospitalizations with or without CLABSI followed a cubic trend (cubic *p*_trend_ = 0.003 and <0.001, respectively), which did differ between those with or without CLABSI (cubic interaction *p* = 0.030). Trends were mostly constant from 2016 to 2019, with a drastic increase during the COVID pandemic in 2020 and 2021 (all *p* < 0.001 compared to 2016–2019). Although a decrease is observed in 2022 (*p* < 0.001 relative to 2021), rates did not return to pre-COVID pandemic levels.

Unadjusted and adjusted model results are presented in Table 3 and Figure 2. Overall, the adjusted model had strong discriminant ability as Harrell’s c-statistic was 0.81. All common health disparities and leading health indicators were associated with in-hospital mortality. Females had 11.3% lower adjusted odds compared to males (95% CI: 10.2% to 12.4%, *p* < 0.001). When compared to Whites, Hispanics had 25.7% greater adjusted odds (95% CI: 22.7% to 28.8%, *p* < 0.001) and Blacks had 2.3% greater adjusted odds (95% CI: 1.01 to 1.04, *p* = 0.022). The lowest income quartile was associated with 12.1% greater adjusted odds compared to the highest income quartile (95% CI: 9.7% to 14.6%, *p* < 0.001). Living in a rural zip code was associated with 7.1% greater odds (95% CI: 4.6% to 9.6%, *p* < 0.001). When compared to privately insured, Medicaid beneficiaries had 6.8% greater adjusted odds (95% CI: 4.4% to 9.2%, *p* < 0.001), whereas Medicare beneficiaries had 3.9% lower adjusted odds (95% CI: 2.1% to 5.7%, *p* < 0.001). Further, alcohol abuse was associated with 12.5% higher adjusted odds of in-hospital death (95% CI: 9.9% to 15.1%, *p* < 0.001). For covariates, higher adjusted odds of in-hospital mortality were associated with older age, absence of a health-related social need, being treated at an urban teaching hospital, having additional CVCs placed during the hospital stay, having the CVC placed in the ICU, and having a greater comorbidity burden.

Although the overall adjusted association between CLABSI and in-hospital mortality was not statistically significant (adjusted odds ratio [aOR]: 0.91, 95% CI: 0.80–1.02, *p* = 0.114), this association differed across primary payers (interaction *p* = 0.025) and alcohol abuse (interaction *p* < 0.001; Table 4). Specifically, the adjusted association between CLABSI and in-hospital mortality in Medicaid beneficiaries was statistically different from the adjusted odds in privately insured (*p* = 0.008) and Medicare beneficiaries (*p* = 0.026); the adjusted odds did not differ between privately insured and Medicare beneficiaries (*p* = 0.387). The sensitivity analysis that omitted hospitalizations in which the patient was transferred to another acute care facility produced nearly identical results to those reported above (Supplemental Tables S3 and S4; Supplemental Figure S1).

### 3.4. Length of Stay

The overall LOS during the study period was 8.7 days (95% CI: 8.6 to 8.7 days). Unadjusted and adjusted model results are presented in Supplemental Tables S5 and S6 and Supplemental Figure S2. Briefly, longer adjusted LOS was observed in males (adjusted ratio [aRatio]: 1.05, 95% CI: 1.04–1.05, *p* < 0.001), Blacks (aRatio: 1.08, 95% CI: 1.08–1.09, *p* < 0.001) and Hispanics (aRatio: 1.07, 95% CI: 1.06–1.08, *p* < 0.001) compared to Whites, living in a rural zip code (aRatio: 1.03, 95% CI: 1.02–1.04, *p* < 0.001), and Medicaid beneficiaries compared to privately insured (aRatio: 1.07, 95% CI: 1.06–1.07, *p* < 0.001). CLABSI was associated with 97.4% longer adjusted LOS (95% CI: 92.3% to 102.6%, *p* < 0.001), but this effect differed by race (interaction *p* = 0.006), primary payer (interaction *p* = 0.009), and in those with a housing-specific health-related social need (interaction *p* = 0.006). Specifically, the adjusted association between CLABSI and LOS was larger in Whites and Hispanics compared to Blacks (*p* = 0.003 and *p* = 0.003, respectively), in Medicaid beneficiaries and privately insured compared to Medicare beneficiaries (*p* = 0.008 and *p* = 0.048, respectively).

### 3.5. Inflation-Adjusted Hospital Cost

The overall inflation-adjusted hospital cost during the study period was $29,694 (95% CI: $29,483 to $29,906). Unadjusted and adjusted model results are presented in Supplemental Tables S7 and S8 and Supplemental Figure S3. Briefly, greater adjusted hospital cost was observed in males (aRatio: 1.10, 95% CI: 1.10–1.10, *p* < 0.001), Blacks (aRatio: 1.04, 95% CI: 1.03–1.05, *p* < 0.001) and Hispanics (aRatio: 1.19, 95% CI: 1.18–1.20, *p* < 0.001) compared to Whites, the highest income quartile compared to the lowest income quartile (aRatio: 1.22, 95% CI: 1.20–1.23, *p* < 0.001), living in a rural zip code (aRatio: 1.05, 95% CI: 1.04–1.07, *p* < 0.001), and Medicaid beneficiaries compared to privately insured (aRatio: 1.02, 95% CI: 1.01–1.02, *p* < 0.001). CLABSI was associated with 82.3% higher adjusted hospital cost (95% CI: 76.7% to 88.0%, *p* < 0.001), but this effect differed by race (interaction *p* = 0.006), with the effect of CLABSI being larger in Whites and Hispanics compared to Blacks (*p* = 0.002 and *p* < 0.001, respectively).

## 4. Discussion

In this large-scale investigation of the NIS database, which represents over 35 million hospitalizations annually across 97% of the U.S. population when weighted [17], approximately 11.5 million CVCs were placed between 2016 and 2022. Among CLABSI-eligible CVCs (as defined by the AHRQ [18]), 1 in 400 (0.25%) were complicated by CLABSI. Consistent with prior studies on racial disparities in hospitalized-acquired infections, we found that Blacks had nearly 30% higher odds of developing CLABSI than Whites, a difference that remained statistically significant after adjusting for covariates [26,27,28,29]. Sex, income quartile, and home community size, however, did not impact CLABSI risk. This suggests that racial disparities in CLABSI may be driven by factors beyond socioeconomic status, such as hospital-specific variations in catheter care, implicit bias, or differences in comorbidities [30,31]. In this regard, the differences in CLABSI rates by hospital type and geography may reflect variability in line utilization, staffing ratios, greater procedural burden and care complexity, and adherence to infection prevention protocols.

Although 14.1% of hospitalizations with CLABSI resulted in in-hospital mortality, CLABSI did not independently increase mortality risk in our adjusted analysis. The lack of an independent association between CLABSI and in-hospital mortality after adjustment may reflect the influence of confounding clinical factors that drive both infection risk and mortality—such as comorbidity burden, ICU-level interventions, and length of stay. It is also possible that some deaths attributed to CLABSI in unadjusted analyses are more accurately attributable to underlying critical illness severity captured in our multivariable models. This contrasts with prior studies [32,33], likely due to our large sample size and the limitation of associating, and not directly attributing, mortality to CLABSI. Subgroup interaction analyses of sex, race, socioeconomic status, and community size revealed no significant disparities in CLABSI-associated mortality, possibly reflecting the impact of standardized hospital protocols for CLABSI events. However, differences emerged based on payer type, with Medicaid beneficiaries with CLABSI showing significantly higher odds of mortality compared to those with private insurance, which may suggest structural inequities in access to advanced care.

CLABSI rates remained stable from 2016 to 2019, surged during the COVID-19 pandemic, and returned to pre-pandemic levels in 2022. This trend aligns with prior studies linking increased ICU admissions, prolonged CVC dwell times, and strained hospital resources to higher nosocomial infection rates during the pandemic [34,35,36,37,38]. Although CLABSI rates generally declined with age, mortality among those who developed CLABSI increased, suggesting greater vulnerability to severe complications in older adults. This paradoxical trend underscores the need for further research into age-related differences in CLABSI risk, management, and outcomes. Furthermore, CLABSI was associated with a 97% longer hospital LOS and an 82% increase in hospitalization costs. Although the impact on LOS has been previously documented [39], the resulting economic burden is less studied. This analysis of the NIS is particularly robust given its ability to capture hospitalization costs at a national level, providing more accurate and generalizable cost estimates than local or single-center studies, which often lack comprehensive financial data.

Our findings build upon and complement those of Tripathi et al. who conducted a multicenter study across 15 hospitals using detailed electronic health record (EHR) data to assess CLABSI rates by race and ethnicity [29]. Their work, while highly granular—benefiting from line-day level exposure data and patient-specific line utilization (e.g., chemotherapy, TPN)—was regionally constrained and included a relatively small number of CLABSI cases (*n* = 263). In contrast, our study offers broader national representation and substantially greater statistical power by leveraging the National Inpatient Sample, which includes over 6.5 million CLABSI-eligible hospitalizations and generates nationally weighted estimates. Although we could not capture catheter-days or some patient-level variables (e.g., primary language), our analysis offers stronger external validity and evaluates disparities across a wider socioeconomic spectrum. Notably, whereas Tripathi et al. found increased CLABSI risk primarily among Hispanic patients, our study identified a statistically significant increased risk among Black patients and Medicaid beneficiaries. Health systems should implement CLABSI prevention strategies targeted toward high-risk populations identified in this study—particularly Black patients and Medicaid beneficiaries—who showed significantly higher adjusted odds of CLABSI.

Future investigations should focus on uncovering the hospital- and provider-level factors that underlie observed disparities in CLABSI incidence—such as variation in line maintenance practices, staff training, and resource allocation. Linking administrative data with electronic health records could enable more granular analysis of catheter-day exposures and real-time risk factors. Additionally, interventional studies are needed to assess the impact of targeted infection prevention programs in high-risk populations, particularly within safety-net hospitals. Policymakers and healthcare systems should also explore the utility of equity-focused quality metrics to drive accountability in infection control practices [40,41].

Several limitations should be considered when interpreting our results. First, the NIS lacks a present-on-admission indicator, requiring the use of AHRQ’s patient safety indicator (PSI 07) to identify hospital-acquired CLABSI [42]. While PSI 07 has been validated for use in administrative datasets, it may undercount true infections by excluding cases with limited documentation or overcount due to misclassification of bloodstream infections unrelated to catheter use. This potential misclassification bias could attenuate or exaggerate observed disparities and associations, particularly across hospitals with variable coding practices or documentation quality. For example, misclassification that disproportionately affects marginalized populations may lead to underestimation of the disparities observed in our study, whereas misclassification from contamination or unrelated infections could bias results in either direction. Second, reliance on administrative coding may introduce bias due to variable coding practices across hospitals, particularly in resource-limited settings. Third, we could not assess hospital-level factors, such as nurse staffing ratios or infection control protocols, which may influence CLABSI risk. Lastly, despite adjusting for multiple confounders, residual confounding from unmeasured factors remains possible. Future research should explore hospital-level interventions and policy changes to mitigate CLABSI disparities and improve patient outcomes.

## 5. Conclusions

This large-scale retrospective study identified significant racial and socioeconomic disparities in the incidence of CLABSI among hospitalized adults in the United States. Black patients and Medicaid beneficiaries exhibited notably higher odds of developing CLABSI compared to their White and privately insured counterparts, respectively. Although CLABSI did not independently increase adjusted in-hospital mortality, it significantly increased hospital length of stay and healthcare costs, imposing substantial clinical and economic burdens. Our findings underscore the necessity for targeted interventions and equity-focused healthcare policies aimed at mitigating these disparities. Further research should explore hospital-level factors and prevention strategies specifically tailored to vulnerable populations to reduce CLABSI rates and associated healthcare utilization.

## Figures and Tables

**Table 1:** Demographic and clinical characteristics.

	Statistic	Range
**Hospitalizations Meeting Inclusion**, count		
Unweighted	1,311,933	
Weighted	6,559,663	
**CVC Count**, %		
1	90.9	
2	7.8	
3+	1.3	
**ICU (Intubation and/or Pressor)**, %	31.2	
Intubation	29.9	
Pressor	3.6	
**CVC Placed in ICU**, %	24.4	
**Total Parenteral Nutrition**, %	2.3	
**Dialysis**, %	7.2	
**Age**, median [IQR]	63 [51–74]	18–90
**Sex**, %		
Male	52.0	
Female	48.0	
**Race**, %		
White	69.3	
Black	15.5	
Hispanic	9.7	
Other	5.6	
**Primary Payer**, %		
Medicare	54.5	
Medicaid	17.2	
Private	21.5	
Other	6.8	
**Income Quartile**, %		
1	32.2	
2	26.4	
3	23.1	
4	18.2	
**Urban/Rural (patient)**, %		
Urban	83.3	
Rural	16.7	
**History of Alcohol Abuse**, %		
No	92.6	
Yes	7.4	
**Health-related Social Needs**, %		
Employment Issue	0.2	
Family Issue	0.2	
Housing Issue	1.1	
Psycho-social Issue	0.1	
Socioeconomic Issue	0.4	
**Charlson Comorbidity Index**, median [IQR]	2 [1–5]	0–18
**Hospital Location-Teaching Status**, %		
Rural	6.7	
Urban Nonteaching	20.9	
Urban Teaching	72.3	
**Year of Hospitalization**, %		
2016	15.7	
2017	15.6	
2018	14.6	
2019	13.8	
2020	13.7	
2021	14.1	
2022	12.3	

Abbreviations: CVC = central venous catheter; ICU = intensive care unit; IQR = interquartile range.

**Table 2:** Unadjusted and adjusted model results for CLABSI.

	Unadjusted			Adjusted	
	%	OR (95% CI)	*p*	OR (95% CI)	*p*
**Overall**	0.25	-	-	-	-
**CVC Count**					
3+	1.75	9.93 (8.73–11.03)	<0.001	8.29 (7.25–9.48)	<0.001
2	0.77	4.37 (4.01–4.76)	<0.001	4.01 (3.66–4.39)	<0.001
1	0.18	Reference		Reference	
**CVC Placed in ICU**					
Yes	0.23	0.94 (0.87–1.03)	0.176	0.68 (0.62–0.74)	<0.001
No	0.25	Reference		Reference	
**Age** (per 10 years)	Figure 2	0.80 (0.79–0.82)	<0.001	0.77 (0.75–0.79)	<0.001
**Sex**					
Female	0.24	0.92 (0.86–0.99)	0.023	0.94 (0.88–1.02)	0.123
Male	0.26	Reference		Reference	
**Race**					
Black	0.36	1.71 (1.56–1.87)	<0.001	1.30 (1.18–1.43)	<0.001
Hispanic	0.29	1.38 (1.22–1.57)	<0.001	1.13 (0.99–1.28)	0.062
Other	0.25	1.20 (1.03–1.40)	0.022	1.06 (0.90–1.24)	0.500
White	0.21	Reference		Reference	
**Primary Payer**					
Medicare	0.20	0.80 (0.73–0.88)	<0.001	1.15 (1.03–1.28)	0.012
Medicaid	0.38	1.51 (1.36–1.68)	<0.001	1.18 (1.06–1.32)	0.002
Other	0.26	1.02 (0.88–1.19)	0.786	0.96 (0.82–1.12)	0.581
Private	0.25	Reference		Reference	
**Income Quartile**					
1	0.28	1.28 (1.14–1.43)	<0.001	1.03 (0.92–1.16)	0.577
2	0.23	1.07 (0.95–1.20)	0.268	0.98 (0.87–1.10)	0.716
3	0.23	1.06 (0.94–1.19)	0.374	0.98 (0.87–1.11)	0.759
4	0.22	Reference		Reference	
**Urban/Rural (patient)**					
Rural	0.23	0.93 (0.84–1.03)	0.187	1.06 (0.94–1.20)	0.350
Urban	0.25	Reference		Reference	
**History of Alcohol Abuse**					
Yes	0.22	0.90 (0.78–1.03)	0.124	0.71 (0.62–0.82)	<0.001
No	0.25	Reference		Reference	
**Health-related Social Needs**					
Employment Issue	*	*	*	*	*
Family Issue	*	*	*	*	*
Housing Issue					
Yes	0.28	1.13 (0.79–1.62)	0.515	0.89 (0.62–1.29)	0.543
No	0.25	Reference		Reference	
Psycho-social Issue	*	*	*	*	*
Socioeconomic Issue					
Yes	0.27	1.11 (0.66–1.86)	0.688	1.10 (0.66–1.81)	0.717
No	0.25	Reference		Reference	
**Charlson Comorbidity Index**	Figure 2	1.08 (1.07–1.09)	<0.001	1.09 (1.08–1.11)	<0.001
**Hospital Location-Teaching Status**					
Rural	0.16	0.58 (0.48–0.69)	<0.001	0.65 (0.53–0.80)	<0.001
Urban Nonteaching	0.17	0.63 (0.57–0.70)	<0.001	0.73 (0.65–0.81)	<0.001
Urban Teaching	0.27	Reference		Reference	
**Year of Hospitalization**					
2016	0.25	1.11 (0.95–1.29)	0.191	1.22 (1.05–1.42)	0.010
2017	0.22	0.98 (0.83–1.15)	0.791	1.05 (0.89–1.23)	0.574
2018	0.20	0.91 (0.77–1.07)	0.250	0.96 (0.82–1.13)	0.625
2019	0.23	1.02 (0.86–1.20)	0.840	1.06 (0.90–1.25)	0.495
2020	0.29	1.32 (1.13–1.53)	<0.001	1.30 (1.12–1.50)	0.001
2021	0.31	1.38 (1.18–1.61)	<0.001	1.34 (1.15–1.56)	<0.001
2022	0.22	Reference		Reference	

*Note.* An * indicates that the results could not be presented due to low observed hospitalization counts per the NIS Data Use Agreement. Abbreviations: CLABSI = central line-associated bloodstream infection; OR = odds ratio; CI = confidence interval; CVC = central venous catheter; ICU = intensive care unit.

**Table 3:** Unadjusted and adjusted model results for in-hospital mortality.

	Unadjusted			Adjusted	
	%	OR (95% CI)	*p*	aOR (95% CI)	*p*
**Overall**	13.3	-	-	-	-
**CLABSI**					
Yes	14.1	1.07 (0.96–1.19)	0.218	0.91 (0.80–1.02)	0.114
No	13.3	Reference		Reference	
**CVC Count**					
3+	31.3	3.37 (3.25–3.49)	<0.001	2.34 (2.24–2.45)	<0.001
2	26.2	2.62 (2.57–2.68)	<0.001	2.02 (1.98–2.06)	<0.001
1	11.9	Reference		Reference	
**CVC Placed in ICU**					
Yes	34.8	7.84 (7.73–7.94)	<0.001	7.20 (7.10–7.30)	<0.001
No	6.4	Reference		Reference	
**Age** (per 10 years)	Figure 2	1.24 (1.24–1.25)	<0.001	1.33 (1.32–1.33)	<0.001
**Sex**					
Female	12.2	0.84 (0.83–0.85)	<0.001	0.89 (0.88–0.90)	<0.001
Male	14.3	Reference		Reference	
**Race**					
Black	12.9	1.01 (0.99–1.03)	0.161	1.02 (1.00–1.04)	0.022
Hispanic	15.7	1.27 (1.23–1.30)	<0.001	1.26 (1.23–1.29)	<0.001
Other	16.6	1.36 (1.32–1.39)	<0.001	1.20 (1.17–1.24)	<0.001
White	12.8	Reference		Reference	
**Primary Payer**					
Medicare	14.9	1.48 (1.45–1.51)	<0.001	0.96 (0.94–0.98)	<0.001
Medicaid	11.0	1.04 (1.01–1.06)	0.002	1.07 (1.04–1.09)	<0.001
Other	14.1	1.38 (1.34–1.42)	<0.001	1.28 (1.25–1.32)	<0.001
Private	10.6	Reference		Reference	
**Income Quartile**					
1	13.9	1.10 (1.07–1.12)	<0.001	1.12 (1.10–1.15)	<0.001
2	13.1	1.03 (1.01–1.05)	0.013	1.06 (1.04–1.08)	<0.001
3	13.0	1.02 (0.99–1.04)	0.081	1.03 (1.01–1.05)	0.005
4	12.8	Reference		Reference	
**Urban/Rural (patient)**					
Rural	13.3	1.01 (0.99–1.03)	0.534	1.07 (1.05–1.10)	<0.001
Urban	13.3	Reference		Reference	
**History of Alcohol Abuse**					
Yes	15.9	1.26 (1.23–1.28)	<0.001	1.12 (1.10–1.15)	<0.001
No	13.1	Reference		Reference	
**Health-related Social Needs**					
Employment Issue					
Yes	6.8	0.48 (0.39–0.57)	<0.001	0.65 (0.54–0.79)	<0.001
No	13.3	Reference		Reference	
Family Issue					
Yes	10.9	0.79 (0.70–0.91)	<0.001	0.82 (0.71–0.94)	0.006
No	13.3	Reference		Reference	
Housing Issue					
Yes	6.8	0.48 (0.44–0.52)	<0.001	0.62 (0.57–0.68)	<0.001
No	13.3	Reference		Reference	
Psycho-social Issue					
Yes	9.1	0.65 (0.51–0.84)	<0.001	0.74 (0.60–0.92)	0.007
No	13.3	Reference		Reference	
Socioeconomic Issue					
Yes	7.8	0.55 (0.50–0.62)	<0.001	0.55 (0.49–0.61)	<0.001
No	13.3	Reference		Reference	
**Charlson Comorbidity Index**	Figure 2	1.12 (1.12–1.12)	<0.001	1.05 (1.05–1.05)	<0.001
**Hospital Location-Teaching Status**					
Rural	11.5	0.81 (0.78–0.84)	<0.001	0.8 (0.77–0.83)	<0.001
Urban Nonteaching	11.9	0.84 (0.82–0.86)	<0.001	0.9 (0.88–0.92)	<0.001
Urban Teaching	13.8	Reference		Reference	
**Year of Hospitalization**					
2016	9.8	0.59 (0.57–0.61)	<0.001	0.68 (0.66–0.71)	<0.001
2017	10.2	0.62 (0.60–0.64)	<0.001	0.68 (0.66–0.71)	<0.001
2018	10.4	0.63 (0.61–0.66)	<0.001	0.68 (0.65–0.70)	<0.001
2019	10.8	0.66 (0.64–0.69)	<0.001	0.68 (0.66–0.70)	<0.001
2020	17.0	1.12 (1.08–1.16)	<0.001	1.05 (1.01–1.09)	0.009
2021	20.4	1.40 (1.36–1.45)	<0.001	1.39 (1.35–1.44)	<0.001
2022	15.5	Reference		Reference	

Abbreviations: OR = odds ratio; aOR = adjusted odds ratio; CI = confidence interval; CLABSI = central line-associated bloodstream infection; CVC = central venous catheter; ICU = intensive care unit.

**Table 4:** Adjusted interaction effects of in-hospital mortality for CLABSI vs. no CLABSI by health disparity and leading health indicator.

	CLABSI vs. No CLABSI		
	aOR (95% CI)	*p*	Interaction *p*
**Sex**			
Female	1.03 (0.85–1.24)	0.777	0.097
Male	0.83 (0.81–0.98)	0.025	
**Race**			
Black	0.76 (0.57–1.01)	0.062	0.085
Hispanic	1.22 (0.92–1.61)	0.163	
Other	0.75 (0.46–1.22)	0.243	
White	0.90 (0.77–1.06)	0.215	
**Primary Payer**			
Medicare	0.97 (0.82–1.16)	0.764	0.025
Medicaid	0.67 (0.52–0.88)	0.004	
Other	0.67 (0.40–1.11)	0.121	
Private	1.12 (0.87–1.44)	0.398	
**Income Quartile**			
1	0.82 (0.66–1.01)	0.062	0.492
2	0.99 (0.78–1.25)	0.939	
3	1.01 (0.79–1.31)	0.919	
4	0.85 (0.63–1.13)	0.261	
**Urban/Rural (patient)**			
Rural	1.12 (0.83–1.50)	0.459	0.130
Urban	0.87 (0.76–0.99)	0.041	
**History of Alcohol Abuse**			
Yes	0.35 (0.20–0.61)	<0.001	<0.001
No	0.97 (0.85–1.10)	0.602	
**Health-related Social Needs**			
Employment Issue			
Yes	*	*	*
No	*	*	
Family Issue			
Yes	*	*	*
No	*	*	
Housing Issue			
Yes	*	*	*
No	*	*	
Psycho-social Issue			
Yes	*	*	*
No	*	*	
Socioeconomic Issue			
Yes	*	*	*
No	*	*	

*Note.* An * indicates that the results could not be presented due to low observed hospitalization counts per the NIS Data Use Agreement. Adjusted odds ratios indicate the odds of in-hospital mortality for hospitalizations that included CLABSI compared to hospitalizations that did not include CLABSI, presented separately by category of a given health disparity or leading health indicators. Abbreviations: CLABSI = central line-associated bloodstream infection; aOR = adjusted odds ratio; CI = confidence interval.

## Data Availability

The data used in this study were obtained from the Healthcare Cost and Utilization Project’s (HCUP) National Inpatient Sample (NIS), sponsored by the Agency for Healthcare Research and Quality (AHRQ). These data are available to researchers through purchase from HCUP (https://www.hcup-us.ahrq.gov/). Restrictions apply to protect patient privacy and confidentiality; therefore, the authors cannot share the dataset directly.

## Supplementary Materials

The following supporting information can be downloaded at: https://www.mdpi.com/article/10.3390/idr17050105/s1, Table S1: ICD-10 diagnosis and procedure codes; Table S2: Descriptive statistics for exclusion criteria; Table S3: In-hospital death excluding transfers—unadjusted/adjusted; Table S4: In-hospital death excluding transfers—adjusted interaction analyses; Table S5: Length of stay—unadjusted and adjusted results; Table S6: Length of stay—adjusted interaction analyses; Table S7: Hospital cost—unadjusted and adjusted results; Table S8: Hospital cost—adjusted interaction analyses; Figure S1: In-hospital death excluding transfers—unadjusted—age and CCI; Figure S2: Length of stay—unadjusted results—age and CCI; Figure S3: Hospital cost—unadjusted results—age and CCI.
