# Supplementary material for: Health Disparities in Central Line-Associated Bloodstream Infections: Analysis of the U.S. National Inpatient Sample Database (2016–2022)
Source: Infect Dis Rep. 2025 Aug 28;17(5):105. doi: 10.3390/idr17050105 (PMC12452301; doi:10.3390/idr17050105)
Supplement: Supplementary file 1 [file idr-17-00105-s001.zip › idr-3750875-supplementary.pdf]

Supplemental Content

Supplemental Table S1. ICD-10 diagnosis and procedure codes 2

Supplemental Table S2. Descriptive statistics for exclusion criteria 4

Supplemental Table S3. In-hospital death excluding transfers – unadjusted/adjusted.................5

Supplemental Figure S1. In-hospital death excluding transfers – unadjusted– age and CCI........7

Supplemental Table S4. In-hospital death excluding transfers – adjusted interaction analyses ...8

Supplemental Table S5. Length of stay – unadjusted and adjusted results..................................9

Supplemental Figure S2. Length of stay – unadjusted results – age and CCI............................11

Supplemental Table S6. Length of stay – adjusted interaction analyses....................................12

Supplemental Table S7. Hospital cost – unadjusted and adjusted results..................................13

Supplemental Figure S3. Hospital cost – unadjusted results – age and CCI..............................15

Supplemental Table S8. Hospital cost – adjusted interaction analyses......................................16

Supplemental Table S1. ICD-10 diagnosis and procedure codes

Diagnosis	ICD-10-CM	Elixhauser
**CLABSI (initial encounter)**	T80211A	
**Exclusion Criteria**		
Cancer	C-, D47Z2-, D47Z9-	
Immunosuppressed	B20, B59, C802, C888, C9440, C9441, C9442, C946, D4622, D4701, D4702, D4709, D471, D479, D47Z1, D47Z2, D47Z9, D6109, D61810, D61811, D61818, D700, D701, D702, D704, D708, D709, D71, D720, D72810, D72818, D72819, D7381, D7581, D761, D762, D763, D800, D801, D802, D803, D804, D805, D806, D807, D808, D809, D810, D811, D812, D8130, D8131, D8132, D8139, D814, D816, D817, D8182, D8189, D819, D820, D821, D822, D823, D824, D828, D829, D830, D831, D832, D838, D839, D840, D841, D848, D8481, D84821, D84822, D8489, D849, D893, D89810, D89811, D89812, D89813, D8982, D8989, D899, E40, E41, E42, E43, I120, I1311, I132, K912, N185, N186, T8600, T8601, T8602, T8603, T8609, T8610, T8611, T8612, T8613, T8619, T8620, T8621, T8622, T8623, T86290, T86298, T8630, T8631, T8632, T8633, T8639, T8640, T8641, T8642, T8643, T8649, T865, T86810, T86811, T86812, T86818, T86819, T86850, T86851, T86852, T86858, T86859, T86890, T86891, T86892, T86898, T86899, T8690, T8691, T8692, T8693, T8699, Z4821, Z4822, Z4823, Z4824, Z48280, Z48288, Z48290, Z48298, Z4901, Z4902, Z4931, Z4932, Z940, Z941, Z942, Z943, Z944, Z9481, Z9482, Z9483, Z9484, Z9489, Z992	
HIV or AIDS	B20	CMR_AIDS
**Covariates**		
Alcohol Abuse		CMR_ALCOHOL
Health-related social needs		
Employment	Z56-, Z57-	
Family	Z62-, Z63-	
Housing	Z590-, Z591-, Z592, Z593	
Psycho-social	Z64-, Z65-	
Socio-economic	Z55-, Z594-, Z595, Z596, Z597-, Z598-, Z599, Z60-	
**Procedure**	**ICD-10-PCS**	
**Central line**	02H633Z, 02HV33Z, 05H533Z, 05H633Z, 05HM33Z, 05HN33Z, 06HM33Z, 06HN33Z	
**Exclusion Criteria**		
Immunosuppressed	02YA0Z0, 02YA0Z2, 0BYC0Z0, 0BYC0Z2, 0BYD0Z0, 0BYD0Z2, 0BYF0Z0, 0BYF0Z2, 0BYG0Z0, 0BYG0Z2, 0BYH0Z0, 0BYH0Z2, 0BYJ0Z0, 0BYJ0Z2, 0BYK0Z0, 0BYK0Z2, 0BYL0Z0, 0BYL0Z2, 0BYM0Z0, 0BYM0Z2, 0DY50Z0, 0DY50Z2, 0DY60Z0, 0DY60Z2, 0DY80Z0, 0DY80Z2, 0DYE0Z0, 0DYE0Z2, 0FY00Z0, 0FY00Z2, 0FYG0Z0, 0FYG0Z2, 0TY00Z0, 0TY00Z2, 0TY10Z0, 0TY10Z2, 0WY20Z0, 0XYJ0Z0, 0XYK0Z0, 30230AZ, 30230G0, 30230G1, 30230G2, 30230G3, 30230G4,	

	30230U2, 30230U3, 30230U4, 30230X0, 30230X1, 30230X2, 30230X3, 30230X4, 30230Y0, 30230Y1, 30230Y2, 30230Y3, 30230Y4, 30233AZ, 30233G0, 30233G1, 30233G2, 30233G3, 30233G4, 30233U2, 30233U3, 30233U4, 30233X0, 30233X1, 30233X2, 30233X3, 30233X4, 30233Y0, 30233Y1, 30233Y2, 30233Y3, 30233Y4, 30240AZ, 30240G0, 30240G1, 30240G2, 30240G3, 30240G4, 30240U2, 30240U3, 30240U4, 30240X0, 30240X1, 30240X2, 30240X3, 30240X4, 30240Y0, 30240Y1, 30240Y2, 30240Y3, 30240Y4, 30243AZ, 30243G0, 30243G1, 30243G2, 30243G3, 30243G4, 30243U2, 30243U3, 30243U4, 30243X0, 30243X1, 30243X2, 30243X3, 30243X4, 30243Y0, 30243Y1, 30243Y2, 30243Y3, 30243Y4, 3E03005, 3E0300P, 3E030U1, 3E030WL, 3E03305, 3E0330P, 3E033U1, 3E033WL, 3E04005, 3E0400P, 3E040WL, 3E04305, 3E0430P, 3E043WL, 3E0A305, 3E0J3U1, 3E0J7U1, 3E0J8U1, XW01318, XW01348, XW03336, XW03351, XW03358, XW03368, XW03378, XW03387, XW03388, XW033B3, XW033C6, XW033D6, XW033H7, XW033J7, XW033K7, XW033M7, XW033N7, XW033S5, XW04336, XW04351, XW04358, XW04368, XW04378, XW04387, XW04388, XW043B3, XW043C6, XW043D6, XW043H7, XW043J7, XW043K7, XW043M7, XW043N7, XW043S5, XW133B8, XW133C8, XW143B8, XW143C8, XW23346, XW23376, XW24346, XW24376	
Chemotherapy	3E04302, 3E04303, 3E04305, 3E0430M, 3E0430P, XW04336, XW04351, XW04358, XW04368, XW04378, XW04387, XW04388, XW043A7, XW043B3, XW043B7, XW043C3, XW043C7, XW043D6, XW043G7, XW043H7, XW043J7, XW043K7, XW043L7, XW043M7, XW043N7, XW043P9, XW043Q5, XW043S5	
**Covariates**		
Vasopressor Use	3E043XZ	
Invasive Mechanical Ventilation	5A1935Z, 5A1945Z, 5A1955Z	
Total Parenteral Nutrition	3E0436Z	
Hemodialysis	5A1D-	

Supplemental Table S2. Descriptive statistics for exclusion criteria

	Statistic
**Hospitalizations with CVC**, count	
Unweighted	2,302,921
Weighted	11,514,602
**Exclusion Criteria (AHRQ)**, %	43.0
CVC on Admission	4.8
CLABSI on Admission	1.0
LOS < 2 Days	4.3
Cancer	14.4
Immunosuppressed	29.2
MS-DRG Ungroupable	0.2
**Hospitalizations Meeting Inclusion**, count	
Unweighted	1,311,933
Weighted	6,559,663

Supplemental Table S3. In-hospital death excluding transfers – unadjusted and adjusted

	Unadjusted			Adjusted	
	**%**	**OR (95% CI)**	**p**	**aOR (95% CI)**	**p**
**Overall**	13.8	-	-	-	-
**CLABSI**					
Yes	14.6	1.07 (0.96-1.19)	0.201	0.90 (0.80-1.02)	0.096
No	13.8	Reference		Reference	
**CVC Count**					
3+	32.7	3.45 (3.33-3.58)	<.001	2.38 (2.28-2.49)	<.001
2	27.3	2.67 (2.62-2.72)	<.001	2.05 (2.00-2.09)	<.001
1	12.3	Reference		Reference	
**CVC Placed in ICU**					
Yes	36.8	8.29 (8.18-8.40)	<.001	7.59 (7.48-7.70)	<.001
No	6.6	Reference		Reference	
**Age** (per 10 years)	Figure S1	1.24 (1.23-1.24)	<.001	1.32 (1.31-1.33)	<.001
**Sex**					
Female	12.6	0.83 (0.82-0.84)	<.001	0.88 (0.87-0.89)	<.001
Male	14.8	Reference		Reference	
**Race**					
Black	13.4	1.01 (0.99-1.03)	0.268	1.02 (0.99-1.04)	0.020
Hispanic	16.2	1.27 (1.24-1.31)	<.001	1.26 (1.23-1.30)	<.001
Other	17.3	1.37 (1.33-1.41)	<.001	1.22 (1.18-1.25)	<.001
White	13.2	Reference		Reference	
**Primary Payer**					
Medicare	15.4	1.46 (1.44-1.49)	<.001	0.94 (0.92-0.96)	<.001
Medicaid	11.4	1.03 (1.01-1.06)	0.004	1.05 (1.02-1.07)	<.001
Other	14.7	1.38 (1.35-1.43)	<.001	1.27 (1.24-1.31)	<.001
Private	11.1	Reference		Reference	
**Income Quartile**					
1	14.4	1.10 (1.07-1.12)	<.001	1.10 (1.08-1.13)	<.001
2	13.6	1.03 (1.01-1.05)	0.007	1.05 (1.03-1.07)	<.001
3	13.5	1.02 (0.99-1.04)	0.084	1.02 (0.99-1.04)	0.023
4	13.3	Reference		Reference	
**Urban/Rural (patient)**					
Rural	14.0	1.03 (1.01-1.05)	0.006	1.07 (1.04-1.09)	<.001
Urban	13.7	Reference		Reference	
**Alcohol Abuse**					
Yes	16.7	1.28 (1.26-1.31)	<.001	1.13 (1.10-1.15)	<.001
No	13.5	Reference		Reference	
**Health-related Social Needs**					
Employment Issue					
Yes	7.0	0.47 (0.39-0.57)	<.001	0.64 (0.53-0.77)	<.001
No	13.8	Reference		Reference	
Family Issue					
Yes	11.2	0.79 (0.69-0.91)	<.001	0.81 (0.70-0.94)	0.005
No	13.8	Reference		Reference	
Housing Issue					
Yes	7.1	0.47 (0.44-0.51)	<.001	0.61 (0.56-0.66)	<.001
No	13.8	Reference		Reference	
Psycho-social Issue					
Yes	9.5	0.66 (0.52-0.84)	<.001	0.73 (0.59-0.91)	0.006
No	13.8	Reference		Reference	
Socioeconomic Issue					

Yes	8.1	0.55 (0.49-0.61)	<.001	0.54 (0.48-0.60)	<.001
No	13.8	Reference		Reference	
**Charlson Comorbidity Index**	Figure S1	1.12 (1.12-1.12)	<.001	1.05 (1.05-1.05)	<.001
**Hospital Location-Teaching Status**					
Rural	12.6	0.87 (0.84-0.91)	<.001	0.91 (0.88-0.95)	<.001
Urban Nonteaching	12.5	0.86 (0.84-0.88)	<.001	0.93 (0.91-0.95)	<.001
Urban Teaching	14.2	Reference		Reference	
**Year of Hospitalization**					
2016	10.1	0.59 (0.57-0.61)	<.001	0.68 (0.66-0.70)	<.001
2017	10.5	0.62 (0.60-0.64)	<.001	0.68 (0.66-0.71)	<.001
2018	10.8	0.63 (0.61-0.66)	<.001	0.68 (0.65-0.70)	<.001
2019	11.2	0.66 (0.64-0.69)	<.001	0.68 (0.66-0.71)	<.001
2020	17.7	1.13 (1.09-1.17)	<.001	1.06 (1.02-1.10)	0.003
2021	21.2	1.41 (1.36-1.45)	<.001	1.41 (1.36-1.46)	<.001
2022	16.0	Reference		Reference	

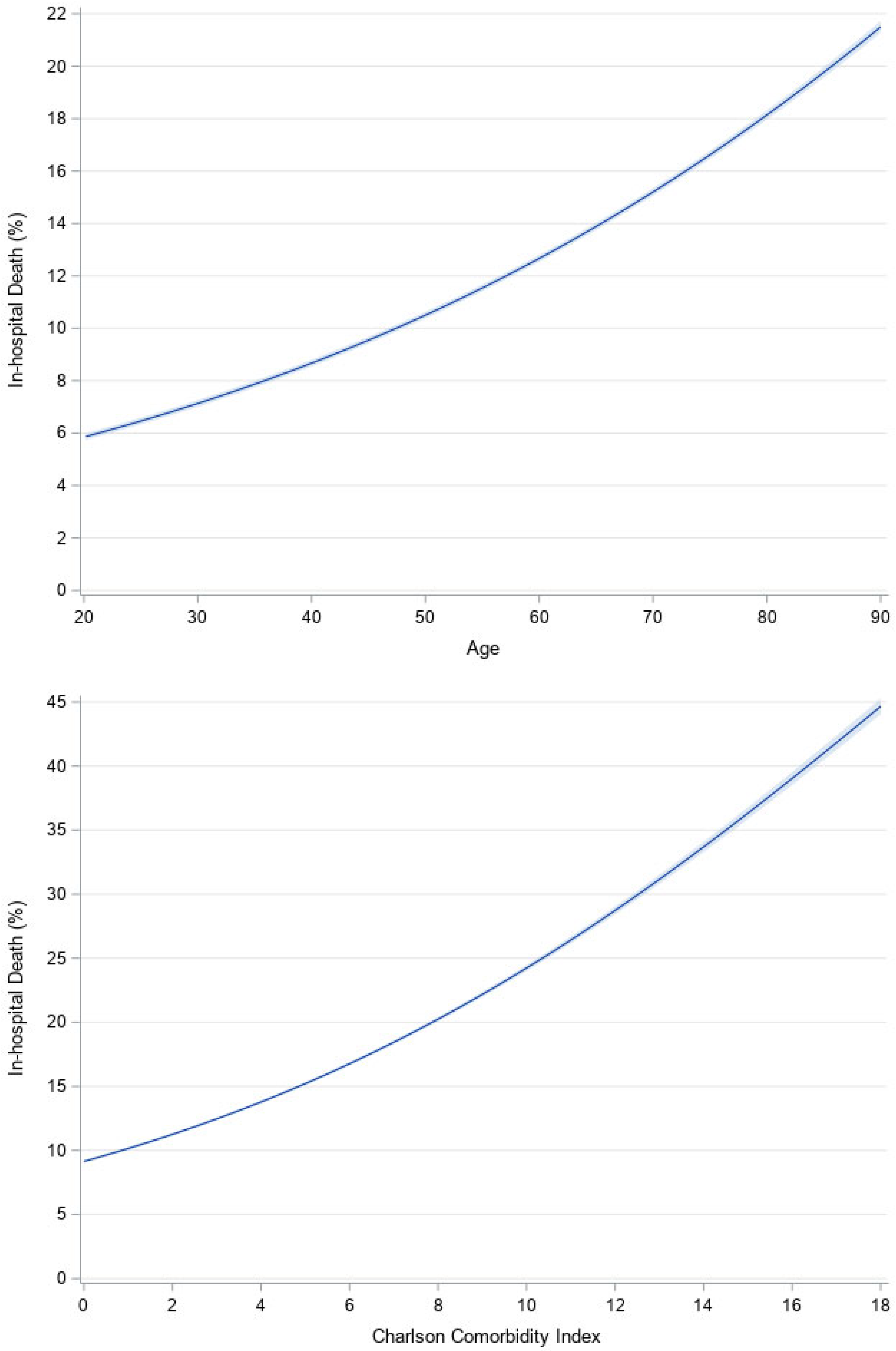

Supplemental Figure S1. In-hospital death excluding transfers – unadjusted results – age and CCI. Shaded areas represent 95% confidence intervals.

Supplemental Table S4. In-hospital death excluding transfers – adjusted interaction analyses

	CLABSI vs. No CLABSI		
	**aOR (95% CI)**	**p**	**Interaction p**
**Sex**			
Female	1.02 (0.85-1.23)	0.817	0.094
Male	0.83 (0.70-0.97)	0.020	
**Race**			
Black	0.73 (0.54-0.97)	0.031	0.056
Hispanic	1.24 (0.93-1.65)	0.149	
Other	0.74 (0.45-1.22)	0.238	
White	0.91 (0.77-1.06)	0.229	
**Primary Payer**			
Medicare	0.97 (0.82-1.15)	0.730	0.022
Medicaid	0.65 (0.50-0.86)	0.003	
Other	0.69 (0.41-1.15)	0.150	
Private	1.12 (0.86-1.45)	0.405	
**Income Quartile**			
1	0.81 (0.65-1.01)	0.052	0.566
2	0.96 (0.76-1.22)	0.751	
3	1.01 (0.78-1.30)	0.955	
4	0.88 (0.65-1.18)	0.377	
**Urban/Rural (patient)**			
Rural	1.10 (0.81-1.48)	0.537	0.155
Urban	0.87 (0.86-0.99)	0.037	
**Alcohol Abuse**			
Yes	0.34 (0.19-0.59)	<.001	<.001
No	0.96 (0.85-1.09)	0.548	
**Health-related Social Needs**			
Employment Issue			
Yes	*	*	*
No	*	*	
Family Issue			
Yes	*	*	*
No	*	*	
Housing Issue			
Yes	*	*	*
No	*	*	
Psycho-social Issue			
Yes	*	*	*
No	*	*	
Socioeconomic Issue			
Yes	*	*	*
No	*	*	

Note. An * indicates that the result cannot be presented due to low unweighted hospitalization counts per the NIS Data Use Agreement.

Supplemental Table S5. Length of stay – unadjusted and adjusted results

	Unadjusted			Adjusted	
	**Median**	**Ratio (95% CI)**	**p**	**aRatio (95% CI)**	**p**
**Overall**	8.7	-	-	-	-
**CLABSI**					
Yes	21.2	2.45 (2.38-2.52)	<.001	1.97 (1.92-2.03)	<.001
No	8.7	Reference			
**CVC Count**					
3+	22.6	2.74 (2.70-2.78)	<.001	2.41 (2.38-2.44)	<.001
2	13.7	1.67 (1.65-1.68)	<.001	1.55 (1.54-1.56)	<.001
1	8.2	Reference		Reference	
**CVC Placed in ICU**					
Yes	9.7	1.16 (1.15-1.16)	<.001	1.04 (1.04-1.05)	<.001
No	8.4	Reference		Reference	
**Age** (per 10 years)	Figure S2	0.99 (0.99-0.99)	<.001	0.99 (0.99-0.99)	<.001
**Sex**					
Female	8.4	0.94 (0.93-0.94)	<.001	0.96 (0.95-0.96)	<.001
Male	9.0	Reference		Reference	
**Race**					
Black	9.5	1.14 (1.13-1.14)	<.001	1.08 (1.08-1.09)	<.001
Hispanic	9.3	1.11 (1.10-1.12)	<.001	1.07 (1.06-1.08)	<.001
Other	9.3	1.11 (1.10-1.12)	<.001	1.07 (1.06-1.08)	<.001
White	8.4	Reference		Reference	
**Primary Payer**					
Medicare	8.5	1.02 (1.01-1.02)	<.001	0.99 (0.98-0.99)	<.001
Medicaid	9.4	1.12 (1.11-1.13)	<.001	1.07 (1.06-1.07)	<.001
Other	9.0	1.07 (1.06-1.08)	<.001	1.04 (1.03-1.05)	<.001
Private	8.4	Reference		Reference	
**Income Quartile**					
1	8.9	1.04 (1.03-1.04)	<.001	1.01 (0.99-1.01)	0.100
2	8.6	1.00 (0.99-1.01)	0.296	1.00 (0.99-1.01)	0.277
3	8.6	1.01 (1.00-1.01)	0.034	1.00 (0.99-1.01)	0.063
4	8.6	Reference		Reference	
**Urban/Rural (patient)**					
Rural	8.3	0.95 (0.94-0.96)	<.001	1.03 (1.02-1.04)	<.001
Urban	8.7	Reference		Reference	
**Alcohol Abuse**					
Yes	9.6	1.12 (1.11-1.12)	<.001	1.04 (1.04-1.05)	<.001
No	8.6	Reference		Reference	
**Health-related Social Needs**					
Employment Issue					
Yes	8.9	1.02 (0.99-1.06)	0.183	0.98 (0.94-1.01)	0.163
No	8.7	Reference		Reference	
Family Issue					
Yes	9.0	1.04 (1.00-1.07)	0.030	1.01 (0.98-1.04)	0.726
No	8.7	Reference		Reference	
Housing Issue					
Yes	10.4	1.20 (1.18-1.22)	<.001	1.16 (1.14-1.18)	<.001
No	8.7	Reference		Reference	
Psycho-social Issue					
Yes	9.3	1.07 (1.02-1.12)	0.004	1.02 (0.97-1.06)	0.473
No	8.7	Reference		Reference	
Socioeconomic Issue					

Yes	9.6	1.11 (1.07-1.14)	<.001	1.08 (1.06-1.11)	<.001
No	8.7	Reference		Reference	
**Charlson Comorbidity Index**	Figure S2	1.05 (1.05-1.05)	<.001	1.04 (1.04-1.04)	<.001
**Hospital Location-Teaching Status**					
Rural	7.2	0.79 (0.78-0.80)	<.001	0.80 (0.79-0.81)	<.001
Urban Nonteaching	7.8	0.86 (0.86-0.87)	<.001	0.89 (0.88-0.89)	<.001
Urban Teaching	9.1	Reference		Reference	
**Year of Hospitalization**					
2016	8.2	0.88 (0.87-0.89)	<.001	0.93 (0.92-0.94)	<.001
2017	8.2	0.88 (0.87-0.90)	<.001	0.92 (0.91-0.93)	<.001
2018	8.3	0.89 (0.88-0.90)	<.001	0.92 (0.91-0.93)	<.001
2019	8.4	0.90 (0.89-0.92)	<.001	0.92 (0.91-0.94)	<.001
2020	9.1	0.97 (0.96-0.99)	<.001	0.97 (0.96-0.98)	<.001
2021	9.6	1.04 (1.02-1.05)	<.001	1.03 (1.02-1.05)	<.001
2022	9.3	Reference		Reference	

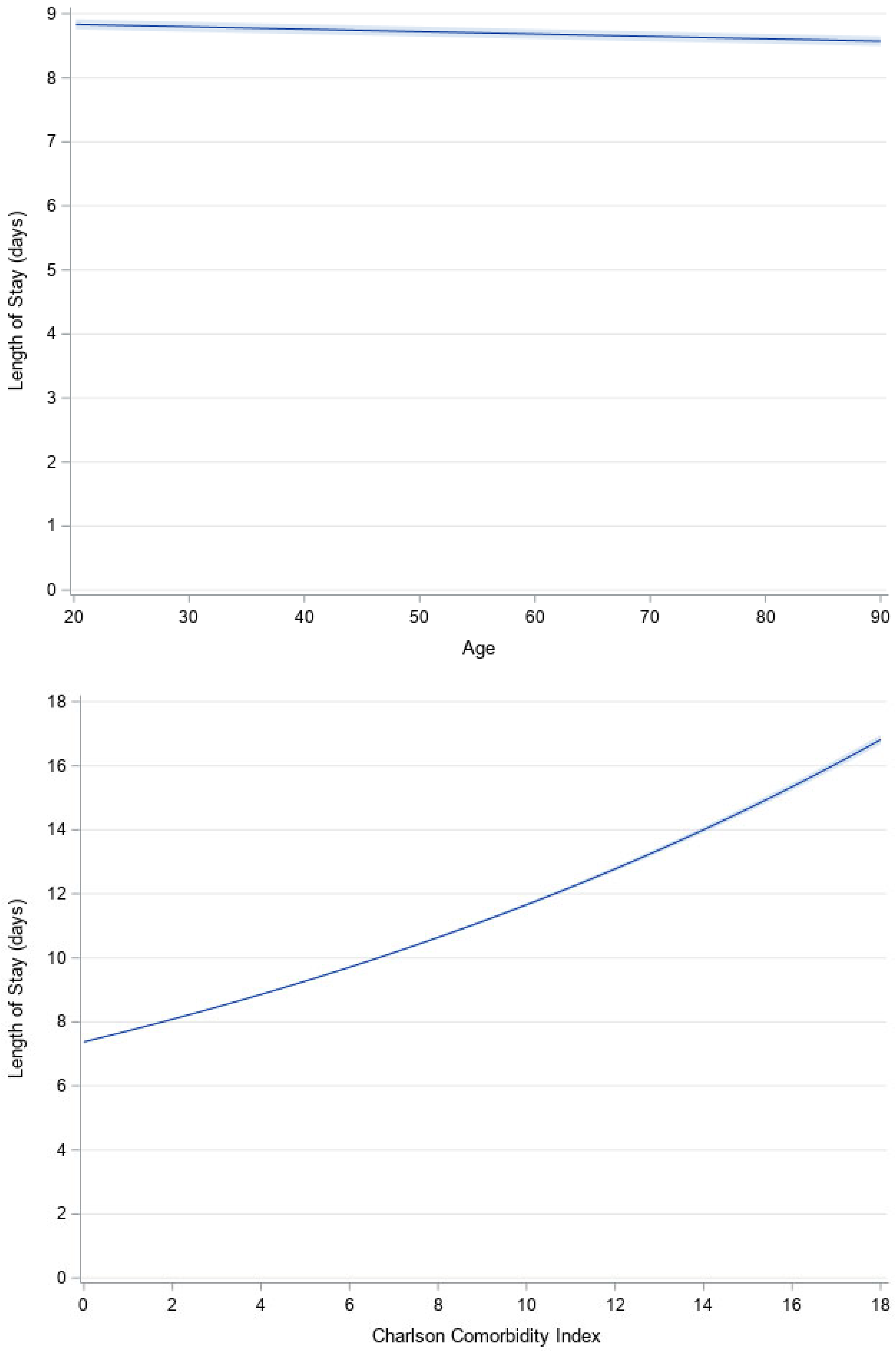

Supplemental Figure S2. Length of stay – unadjusted results – age and CCI. Shaded areas represent 95% confidence intervals.

Supplemental Table S6. Length of stay – adjusted interaction analyses

	CLABSI vs. No CLABSI		
	**aRatio (95% CI)**	**p**	**Interaction p**
**Sex**			
Female	1.94 (1.87-2.02)	<.001	0.264
Male	2.00 (1.93-2.07)	<.001	
**Race**			
Black	1.83 (1.74-1.93)	<.001	0.006
Hispanic	2.12 (1.96-2.29)	<.001	
Other	1.93 (1.72-2.15)	<.001	
White	2.01 (1.94-2.08)	<.001	
**Primary Payer**			
Medicare	1.89 (1.82-1.96)	<.001	0.009
Medicaid	2.05 (1.95-2.17)	<.001	
Other	2.12 (1.94-2.32)	<.001	
Private	2.02 (1.91-2.13)	<.001	
**Income Quartile**			
1	1.91 (1.83-1.99)	<.001	0.211
2	2.01 (1.91-2.12)	<.001	
3	1.99 (1.89-2.10)	<.001	
4	2.05 (1.92-2.18)	<.001	
**Urban/Rural (patient)**			
Rural	1.91 (1.78-2.05)	<.001	0.310
Urban	1.99 (1.93-2.04)	<.001	
**Alcohol Abuse**			
Yes	2.03 (1.86-2.21)	<.001	0.531
No	1.97 (1.92-2.02)	<.001	
**Health-related Social Needs**			
Employment Issue			
Yes	1.81 (0.88-3.72)	0.106	0.814
No	1.97 (1.92-2.03)	<.001	
Family Issue			
Yes	2.49 (1.37-4.53)	0.003	0.442
No	1.97 (1.92-2.03)	<.001	
Housing Issue			
Yes	2.80 (2.18-3.59)	<.001	0.006
No	1.97 (1.92-2.02)	<.001	
Psycho-social Issue			
Yes	2.76 (1.03-7.36)	0.043	0.504
No	1.97 (1.92-2.03)	<.001	
Socioeconomic Issue			
Yes	1.98 (1.41-2.79)	<.001	0.981
No	1.97 (1.92-2.03)	<.001	

Supplemental Table S7. Hospital cost – unadjusted and adjusted results

	Unadjusted			Adjusted	
	**Median**	**Ratio (95% CI)**	**p**	**aRatio (95% CI)**	**p**
**Overall**	29,694	-	-	-	-
**CLABSI**					
Yes	68,819	2.32 (2.24-2.41)	<.001	1.82 (1.77-1.88)	<.001
No	29,632	Reference			
**CVC Count**					
3+	98,531	3.56 (3.50-3.62)	<.001	2.83 (2.78-2.87)	<.001
2	54,421	1.96 (1.94-1.99)	<.001	1.70 (1.69-1.72)	<.001
1	27,709	Reference		Reference	
**CVC Placed in ICU**					
Yes	45,402	1.75 (1.74-1.76)	<.001	1.55 (1.54-1.55)	<.001
No	25,926	Reference		Reference	
**Age** (per 10 years)	Figure S3	0.99 (0.99-0.99)	<.001	0.99 (0.99-0.99)	<.001
**Sex**					
Female	27,578	0.87 (0.86-0.87)	<.001	0.91 (0.91-0.91)	<.001
Male	31,816	Reference		Reference	
**Race**					
Black	30,166	1.07 (1.06-1.07)	<.001	1.04 (1.03-1.05)	<.001
Hispanic	35,202	1.24 (1.23-1.26)	<.001	1.19 (1.18-1.20)	<.001
Other	38,550	1.36 (1.34-1.38)	<.001	1.24 (1.22-1.26)	<.001
White	28,311	Reference		Reference	
**Primary Payer**					
Medicare	28,490	0.93 (0.92-0.93)	<.001	0.92 (0.92-0.93)	<.001
Medicaid	32,287	1.05 (1.04-1.06)	<.001	1.02 (1.01-1.02)	<.001
Other	30,356	0.99 (0.98-1.01)	0.114	0.96 (0.95-0.97)	<.001
Private	30,679	Reference		Reference	
**Income Quartile**					
1	28,115	0.85 (0.84-0.86)	<.001	0.82 (0.81-0.83)	<.001
2	28,670	0.86 (0.85-0.87)	<.001	0.86 (0.85-0.87)	<.001
3	30,566	0.92 (0.91-0.93)	<.001	0.91 (0.91-0.92)	<.001
4	33,175	Reference		Reference	
**Urban/Rural (patient)**					
Rural	27,442	0.91 (0.90-0.92)	<.001	1.05 (1.04-1.07)	<.001
Urban	30,152	Reference		Reference	
**Alcohol Abuse**					
Yes	35,841	1.23 (1.22-1.23)	<.001	1.07 (1.07-1.08)	<.001
No	29,259	Reference		Reference	
**Health-related Social Needs**					
Employment Issue					
Yes	28,287	0.95 (0.92-0.99)	0.012	0.93 (0.89-0.96)	<.001
No	29,696	Reference		Reference	
Family Issue					
Yes	28,826	0.97 (0.94-1.01)	0.101	0.93 (0.90-0.96)	<.001
No	29,696	Reference		Reference	
Housing Issue					
Yes	29,721	1.00 (0.98-1.02)	0.934	0.98 (0.96-1.00)	0.024
No	29,694	Reference		Reference	
Psycho-social Issue					
Yes	29,412	0.99 (0.94-1.04)	0.725	0.94 (0.89-0.98)	0.009
No	29,694	Reference		Reference	
Socioeconomic Issue					

Yes	29,907	1.01 (0.97-1.05)	0.731	1.01 (0.98-1.04)	0.596
No	29,693	Reference		Reference	
**Charlson Comorbidity Index**	Figure S3	1.05 (1.05-1.05)	<.001	1.04 (1.04-1.04)	<.001
**Hospital Location-Teaching Status**					
Rural	23,159	0.73 (0.71-0.74)	<.001	0.79 (0.77-0.81)	<.001
Urban Nonteaching	25,569	0.81 (0.79-0.82)	<.001	0.85 (0.84-0.86)	<.001
Urban Teaching	31,740	Reference		Reference	
**Year of Hospitalization**					
2016	26,117	0.80 (0.78-0.83)	<.001	0.88 (0.85-0.90)	<.001
2017	26,781	0.82 (0.80-0.85)	<.001	0.88 (0.86-0.90)	<.001
2018	27,175	0.83 (0.81-0.86)	<.001	0.88 (0.86-0.90)	<.001
2019	28,878	0.89 (0.86-0.91)	<.001	0.92 (0.90-0.94)	<.001
2020	33,540	1.03 (1.01-1.06)	0.049	1.01 (0.99-1.04)	0.324
2021	35,365	1.09 (1.06-1.12)	<.001	1.06 (1.04-1.09)	<.001
2022	32,567	Reference		Reference	

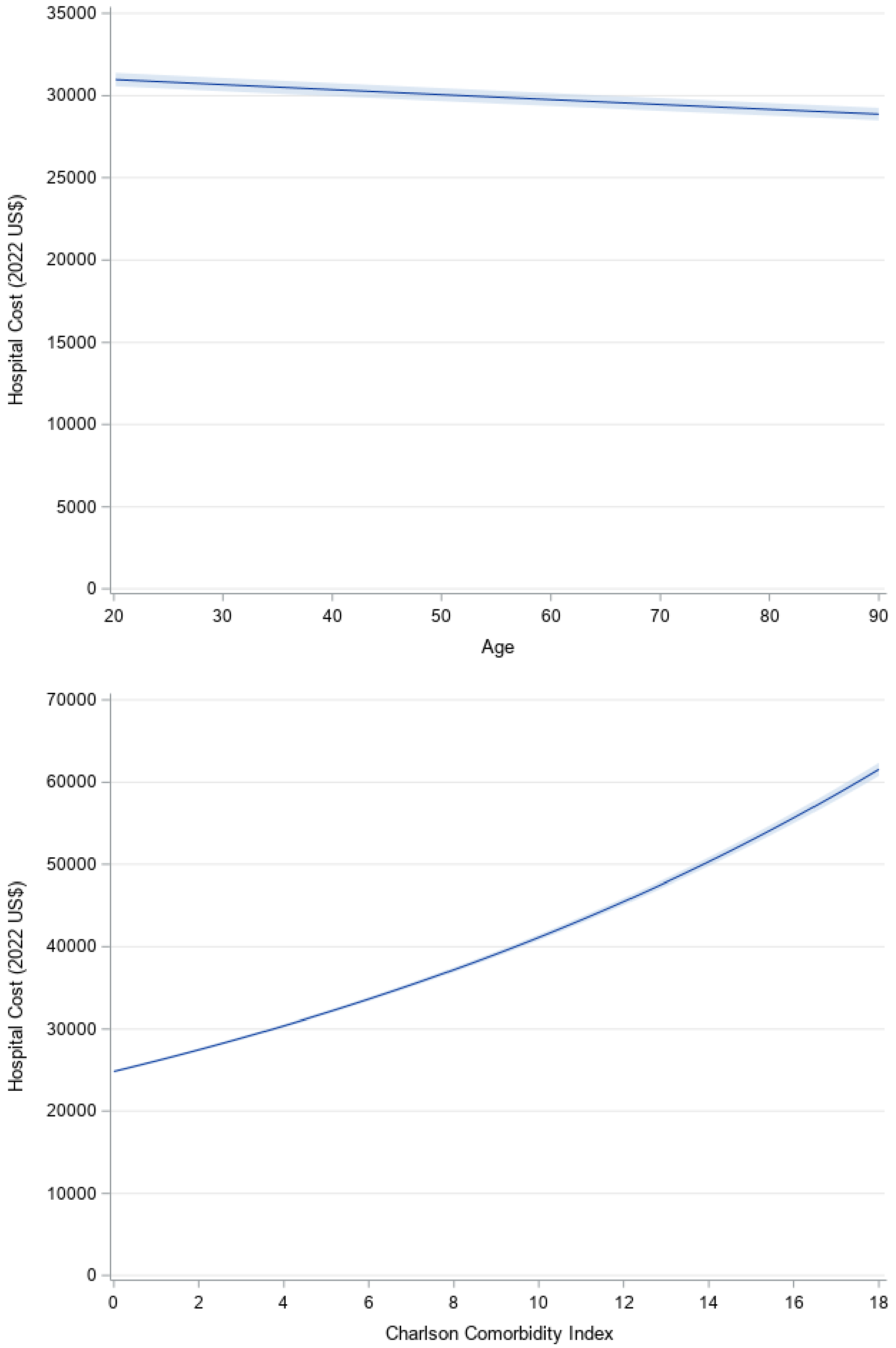

Supplemental Figure S3. Hospital cost – unadjusted results – age and CCI. Shaded areas represent 95% confidence intervals.

Supplemental Table S8. Hospital cost – adjusted interaction analyses

	CLABSI vs. No CLABSI		
	**aRatio (95% CI)**	**p**	**Interaction p**
**Sex**			
Female	1.77 (1.70-1.85)	<.001	0.088
Male	1.87 (1.79-1.95)	<.001	
**Race**			
Black	1.63 (1.53-1.73)	<.001	<.001
Hispanic	2.21 (2.01-2.44)	<.001	
Other	1.86 (1.63-2.13)	<.001	
White	1.83 (1.76-1.90)	<.001	
**Primary Payer**			
Medicare	1.77 (1.70-1.85)	<.001	0.168
Medicaid	1.82 (1.71-1.93)	<.001	
Other	1.84 (1.66-2.04)	<.001	
Private	1.93 (1.81-2.07)	<.001	
**Income Quartile**			
1	1.79 (1.71-1.88)	<.001	0.695
2	1.81 (1.70-1.92)	<.001	
3	1.88 (1.76-2.00)	<.001	
4	1.84 (1.71-1.99)	<.001	
**Urban/Rural (patient)**			
Rural	1.73 (1.60-1.87)	<.001	0.138
Urban	1.84 (1.78-1.90)	<.001	
**Alcohol Abuse**			
Yes	1.86 (1.68-2.07)	<.001	0.666
No	1.82 (1.76-1.88)	<.001	
**Health-related Social Needs**			
Employment Issue			
Yes	1.64 (0.70-3.85)	0.252	0.812
No	1.82 (1.77-1.88)	<.001	
Family Issue			
Yes	2.47 (1.08-5.63)	0.032	0.473
No	1.82 (1.77-1.88)	<.001	
Housing Issue			
Yes	2.13 (1.63-2.80)	<.001	0.253
No	1.82 (1.76-1.88)	<.001	
Psycho-social Issue			
Yes	2.34 (1.28-4.28)	0.006	0.416
No	1.82 (1.77-1.88)	<.001	
Socioeconomic Issue			
Yes	2.06 (1.51-2.80)	<.001	0.440
No	1.82 (1.77-1.88)	<.001	
